# Analysis of excitatory and inhibitory interactions at high temporal resolution in core circuits of the respiratory CPG

**DOI:** 10.1186/1471-2202-13-S1-P39

**Published:** 2012-07-16

**Authors:** Yaroslav I Molkov, Anke Borgmann, Ruli Zhang, Ilya A Rybak, Jeffrey C Smith

**Affiliations:** 1 Department of Mathematical Sciences, Indiana University – Purdue University Indianapolis, IN 46202, USA; 2 Zoological Institute, University of Cologne, Cologne, 50674, Germany; 3 Cellular and Systems Neurobiology Section, NINDS, NIH, Bethesda, MD 20892, USA; 4 Department of Neurobiology and Anatomy, Drexel University College of Medicine, Philadelphia, PA 19129, USA

## 

The pre-Bötzinger complex (pre-BötC) is the essential core component of the brainstem respiratory central pattern generator (rCPG) in mammals. Phasic excitatory and inhibitory synaptic inputs during the respiratory cycle are thought to dynamically shape membrane potential trajectories and spiking patterns of pre-BötC neurons. These synaptic inputs reflect the functionally interacting neuron populations involved in rhythm and inspiratory-expiratory pattern generation. The dynamic patterns of these synaptic inputs have not been systematically studied and characterized experimentally. Accurate temporal reconstruction of the synaptic input patterns is important for testing current models of the rCPG that incorporate specific excitatory and inhibitory circuit mechanisms for rhythm and pattern generation. A recent computational model of the rCPG generating a normal “three-phase” respiratory pattern [1] specified patterns of synaptic excitation and inhibition in the pre-BötC during the respiratory cycle required to produce the behaviorally distinct phases of inspiration (I) and two phases of expiration (E1, or post-inspiration, and E2). This model has not been rigorously tested by experimental data on dynamic changes in phasic postsynaptic excitatory and inhibitory conductances in different populations of pre-BötC inspiratory and expiratory neurons.

We developed analytical techniques that allow quantitative reconstruction of the patterns of synaptic conductances at high temporal resolution from intracellular recordings of membrane potential trajectories. Our approach extends previously proposed methods for extracting dynamic patterns of synaptic input conductances [2,3] and provides nearly continuous readouts of inhibitory and excitatory synaptic conductances in electrophysiologically different cell types throughout the respiratory cycle. The membrane potential trajectories of pre-BötC respiratory neurons were obtained by sharp microelectrode current-clamp recording within *in situ* perfused brainstem-spinal cord preparations of mature rats, which generate a three-phase respiratory pattern and provide mechanically stable conditions for intracellular recordings in functionally intact brainstem circuits.

We distinguished different types of inspiratory and expiratory pre-BötC neurons and analyzed the dynamical changes of excitatory (Ge) and inhibitory (Gi) conductances. Inspiratory neurons exhibited strong excitatory inputs (large dynamic increases in Ge) during their spiking phase followed by a wave(s) of inhibitory inputs during the expiratory period with large increases in Gi and temporal patterns consistent with two populations of inhibitory neurons providing inputs that coordinate inspiratory to expiratory and expiratory to inspiratory phase transitions. Expiratory neurons exhibited strong inhibition during the inspiratory phase, consistent with the rhythmic alternation of inspiration and expiration, and changes in Ge indicating reciprocal interactions between two major populations of inhibitory neurons coordinating the generation of two (E1 and E2) phases of expiration. The reconstructed patterns of Ge and Gi are generally consistent with previously proposed circuit architecture [1] and also suggest its extension. The techniques that we have developed for high temporal resolution of postsynaptic conductance changes are likely to have broad application for analysis of synaptic interactions in circuit components whenever intracellular recordings of membrane potentials can be obtained from functionally intact rhythmically active network.
